# Higher Trait Psychopathy Is Associated with Increased Risky Decision-Making and Less Coincident Insula and Striatal Activity

**DOI:** 10.3389/fnbeh.2017.00245

**Published:** 2017-12-12

**Authors:** Matthew T. Sutherland, Diana H. Fishbein

**Affiliations:** ^1^Department of Psychology, Florida International University, Miami, FL, United States; ^2^Translational Research on Adversity and Neurodevelopment, Department of Human Development and Family Studies, The Pennsylvania State University, University Park, PA, United States

**Keywords:** decision-making, psychopathy, PET, insula, striatum

## Abstract

Higher trait levels of psychopathy have been associated with both a tendency to maintain disadvantageous decision-making strategies and aberrant cortico-limbic neural activity. To explore the neural mechanisms associated with the psychopathy-related propensity to continue selecting risky choices, a non-forensic sample of participants completed a self-report psychopathy questionnaire and two runs of a risky decision-making task during H_2_^15^O positron emission tomography (PET) scanning. In this secondary data analysis study, we leveraged data previously collected to examine the impact of previous drug use on risky decision-making to explore the relations between self-reported psychopathy and behavioral and brain metrics during performance of the Cambridge Decision-Making Task (CDMT), in which volunteers chose between small/likely or large/unlikely potential reward outcomes. Behaviorally, we observed that psychopathy scores were differentially correlated with the percent of risky decisions made in run 1 vs. run 2 of the task. Specifically, higher levels of psychopathy, above and beyond that attributable to drug use or sex, were associated with greater tendencies to make risky selections only in the second half (run 2) of the task. In parallel, psychopathy scores negatively correlated with regional cerebral blood flow (rCBF) in the right insula and right ventral striatum during run 2 of the CDMT. These exploratory outcomes suggest that greater levels of psychopathy may be associated with an inability to translate experience with negative outcomes into behavioral adaptations possibly due to decreased neural efficiency in regions related to somatic and/or reward feedback processes.

## Introduction

Psychopathy is associated with affective and cognitive alterations that are expressed as attenuated emotional responses, impaired moral judgments and impulsive decision-making (Blair, 2005; Kiehl, 2006; Glenn and Raine, 2008; Yang et al., 2008). The behavior of individuals with high levels of psychopathy often enters the realm of criminality given the criminal justice system’s reliance on principles of deterrence, which are based on the ability to anticipate consequences of one’s actions, inhibit responses to avoid punishment, and possess a moral understanding and responsibility for their actions. Even when individuals with psychopathy do not cross the legal boundary or remain undetected by the system, their apparent callousness, emotional detachment, and relative lack of cognitive control are not acceptable in most societal settings, from relationships to the workplace.

Theories abound as to the underpinnings of these traits. Suffice it to say, psychopathy has been repeatedly distinguished by findings of aberrations in psychological, cognitive, psychophysiological, biochemical and neurobiological measures (Hughes et al., 2016). Taken together, these deviations implicate deficits in sensory processing and prediction of internal somatic states that preclude normative emotional experiences, emotion recognition, and fear conditioning that, in turn, perturb decision-making (Marsh, 2013). Criminal psychopaths, for example, show impaired fear conditioning and deficient affect-related information processing that is accompanied by reduced functional activity in amygdala, parahippocampus, insula, orbitofrontal (OFC) and anterior cingulate cortex (ACC; Kiehl et al., 2001; Birbaumer et al., 2005). A reduced capacity for moral reasoning and ethical decision-making in psychopathy has similarly been associated with amygdala and ventromedial prefrontal cortex (vmPFC) dysfunction (Raine and Yang, 2006; Blair, 2007). Furthermore, structural and functional alterations in the OFC, ACC and dorsolateral PFC (dlPFC), possibly underlying poor decision-making and/or impaired cognitive control, have been consistently observed across antisocial populations (Yang and Raine, 2009). Altered functioning of these cortico-limbic regions may underlie maladaptive decision-making resulting from a motivational imbalance characterized by, on the one hand, inattention towards threat and indifference to negative outcomes and, on the other hand, increased impulsivity and heightened reward sensitivity (van Honk and Schutter, 2006).

While exploration of the neural substrates underlying psychopathy has focused on forensic/clinical populations, psychopathic traits lie on a continuum (Miller et al., 2001; Edens et al., 2006). Therefore, assessment of non-forensic samples may be useful to elucidate variability in the pathological state as well as individual differences in brain and behavior. Studies involving non-forensic samples show higher trait levels of psychopathic characteristics in relation to disadvantageous decision-making (van Honk et al., 2002), increased behavioral measures of risk-taking (Hunt et al., 2005), and diminished electrophysiological responses following negative outcomes (Hall et al., 2007). Additionally, several functional imaging and neuroreceptor studies in community volunteers suggest alterations in reward processing, such that higher-trait psychopathy correlates with increased mesolimbic dopamine responsivity to pharmacological and monetary rewards (Buckholtz et al., 2010). Furthermore, albeit among psychopathic offenders, increased striatal gray matter volume (Glenn et al., 2010) and more pronounced cortico-striatal abnormalities among highly impulsive and violent offenders have been documented (Schiffer et al., 2011; Hosking et al., 2017). Thus, consistent with previous studies across a variety of samples, maladaptive decision-making among individuals high in psychopathy may result from reduced cognitive control and neural/somatic *hypo-responsivity* to negative outcomes. On the other hand, dysfunctional or inaccurate representations of reinforcement information may, in effect, lead to increased impulsivity and *hyper-responsivity* to reward (Blair, 2013).

In both forensic and non-forensic participant samples, disadvantageous decision-making generally manifests itself in the latter periods of laboratory-based tasks when participants are expected to shift responses based on feedback. Specifically, during performance of the Iowa Gambling Task, both forensic psychopaths (Mitchell et al., 2002) and individuals high in psychopathic traits (van Honk et al., 2002) continue to make risky selections over the course of the task, whereas comparison individuals tend to decrease such risky choices. The continued selection of disadvantageous options may be indicative of an impaired ability to integrate action outcomes with task contingencies to optimize goal-directed behavior. As such, in the absence of somatic feedback normally associated with losing, an immediate reward becomes more salient (Bechara, 2013). Birbaumer et al. (2005) have provided some indication of the neural substrates related to impaired outcome integration by demonstrating reduced OFC activity in forensic psychopaths, particularly during the latter-half of acquisition in a fear-conditioning paradigm. Other studies have implicated dysfunction within more complex neural circuitry associated with disadvantageous decision-making, with the vmPFC not effectively communicating emotional signals with the amygdala, insula, somatosensory cortices, dlPFC and hippocampus (Bechara, 2004). However, discrepant findings across samples exist and the neural mechanisms mediating the propensity to continue selecting disadvantageous options among non-forensic participants with a range of psychopathic traits remain to be more fully characterized.

Given that antisocial behavior has been linked with prefrontal alterations (Yang and Raine, 2009), decision-making paradigms may be useful probes for the interrogation of cortico-limbic dysfunction in psychopathy. To this end, the present secondary data analysis study considered the relation between self-reported trait psychopathy and behavioral and brain metrics during ongoing performance of a risky decision-making task. The data utilized here were previously collected in a study optimally designed to examine the impact of a drug use history on risky decision-making in a sample of male and female abstinent substance abusers from outpatient treatment programs vs. substance non-abusers from the general population (Fishbein et al., 2005). Acknowledging that the primary study was not optimally designed to examine psychopathy-related influences on risky decision-making, we exerted statistical control over obviously confounding variables (i.e., drug use and sex) and considered any outcomes as exploratory. The utility of such an exploratory, secondary analysis of existing data are the potential contributions to the design of future confirmatory studies to further elucidate psychopathy-related brain alterations in the context of risky decision-making. Participants completed a modified version of the Cambridge Decision-Making Task (CDMT: Rogers et al., 1999b), in which they chose between small/likely or large/unlikely potential reward outcomes, during H_2_^15^O positron emission tomography (PET) scanning. PET images were acquired during performance of two separate task runs and the putative relation between regional cerebral blood flow (rCBF) and self-reported psychopathy was assessed at the whole-brain level. Given that chronic drug use has been related to impaired CDMT performance (Rogers et al., 1999a) and altered functional brain activity during decision-making (Bolla et al., 2003; Ersche et al., 2005; Fishbein et al., 2005), we attempted to statistically control for drug use severity (DUS; as opposed to experimental control at the point of recruitment) in an effort to isolate variability in behavioral and brain measures specifically associated with psychopathy scores above and beyond that attributable to drug use, a confound that plagues many studies of psychopathy (Boccardi et al., 2011). Additionally, most previous studies exploring psychopathy have tended to focus on male participants, thus limiting generalizability. In this secondary data analysis study, both male and female participants were considered and sex was statistically controlled for in the analyses.

Our heuristic framework is based on the premise that an increased propensity to maintain risky decision-making associated with psychopathy is mediated by altered feedback processing such that penalties/errors will be less salient than reward and, thus, risky decisions will not be associated with typical “somatic deterrents” (Bechara and Damasio, 2005). We examined the putative correlations between behavioral and brain metrics during CDMT performance and trait psychopathy when controlling for DUS and sex. We hypothesized that: (1) higher self-reported psychopathy scores would be positively correlated with the percent of risky selections made in the CDMT, where such a relation would become more pronounced as participants experience with the task increased (i.e., during the latter half of the task: Run 2 vs. Run 1); (2) CDMT task performance would be associated with increased rCBF in expected regions including the OFC, insula, and parietal cortex as previously documented in PET studies (Rogers et al., 1999b; Ersche et al., 2005); and (3) higher psychopathy scores would negatively correlate with rCBF in some cognitive control and/or somatic marker processing regions (e.g., OFC, ACC, insula, vmPFC), and/or dlPFC and yet, positively correlate with rCBF in some reward-processing related regions (i.e., striatum).

## Materials and Methods

### Participants

This secondary data analysis study, involved data from 28 individuals (14 females) who participated in a study of the neural substrates of decision-making among abstinent substance abusers vs. non-users (Fishbein et al., 2005); a measure of psychopathy was included in the initial design of this study to test the current hypotheses. Participants were 21–35 years old (26.8 mean ± 0.9 SEM), of average intelligence as determined by the Shipley estimated IQ (102 ± 2), and predominately African American (*n* = 14) and Caucasian (*n* = 11); two were Asian and one Hispanic (Table 1). Individuals over 35 years old were excluded to avoid age-related effects on cognitive parameters (Li et al., 2001). Other exclusionary criteria included: (1) current Axis I diagnoses or current (but not remitted) Axis II diagnoses; (2) current usage of psychotropic or vasoactive medication; (3) a self-reported history of severe head injury (requiring hospitalization or involving more than 3 min of unconsciousness); and (4) left handedness.

**Table 1:** Participant demographic information.

	*N* = 28
Psychopathy score (0–48)	29.5 ± 1.2
Age	26.8 ± 0.9
Sex	14 F, 14 M
IQ (Shipley)	102.2 ± 2.0
Years education	13.9 ± 0.5
Socioeconomic status	3.5 ± 0.2
Race/Ethnicity	14 AA, 11C, 2A, 1H
Drug use severity (all)	3.4 ± 1.0
Number substance abusers	12
Drug use severity (users only)	7.8 ± 1.7

*Note. Sex: F, female; M, male. Race/Ethnicity: AA, African American; C, Caucasian; A, Asian; H, Hispanic. Values reported as mean ± standard error mean*.

Participants included both abstinent substance abusers (*n* = 12) and non-abusers (*n* = 16). Abstinent drug abusers were recruited from outpatient treatment programs in Baltimore City. The investigative staff attended either the first or last several minutes of treatment sessions to describe the study to interested candidates and study flyers were posted in the facilities with contact information. All candidates were routinely urine-screened; participants who were abstinent for a minimum of 3 months were recruited for the study. Abusers reported a regular use history of one or more of the following substances: heroin, cocaine, alcohol, marijuana, hallucinogens, ecstasy, and amphetamines for an average duration of 7.7 ± 4.4 years of abuse. Mean duration of abstinence before data collection was 19 months (range: 3–84 months). A measure of DUS (Fishbein et al., 2005) was calculated for inclusion as a covariate in all analyses. DUS scores consisted of a rating for type of drug (1 = marijuana and hallucinogens; 2 = all other illicit substances and alcohol) that was multiplied by a number assigned to duration ranges (i.e., 0.5 = <1 year, 1 = 1–4 years, 2 = 5–8 years, 3 = 9–12 years, and 4 = ≥13 years). For poly-drug users, scores obtained for individual drugs were summed.

Participants without a history of substance abuse were recruited through advertisements displayed in local newspapers, clinics, and churches within the community where the abstinent abusers largely resided. Non-abusers reported no current or previous history of illicit drug or alcohol abuse. Prior to enrollment, all participants provided written informed consent to a protocol approved by the Institutional Review Board for the National Institute on Drug Abuse-Intramural Research Program (NIDA-IRP). All subjects gave written informed consent in accordance with the Declaration of Helsinki. A “consent test” was administered, including 10 questions about the study procedures and risks. Only those individuals who responded correctly on 80% of the questions were invited to participate and any incorrect responses were carefully reviewed prior to enrollment. In general, all participants were able to understand the study requirements including potential risks and discomforts, provide informed consent, understand task instructions during a carefully managed training session, successfully complete task training, tolerate study procedures, and were treated similarly during study visits (e.g., amount of compensation).

### Procedures

Participants initially visited the NIDA-IRP facility for a neurological exam, urine toxicology screen, a pregnancy test if female, and cardiac evaluation. During this visit, a self-report measure assessing trait-levels of psychopathy (Levenson et al., 1995) and a demographics questionnaire were completed. Additional measures collected during this visit included the: Diagnostic Interview Schedule (DSM-IV criteria: American Psychiatric Association, 1994), Addiction Severity Inventory (McLellan et al., 1992), Symptom Checklist 90-Revised (Derogatis et al., 1973) and Dysregulation Inventory (Mezzich et al., 2001). Participants also underwent a carefully managed mock scanner session to become familiar with the scanner environment, the task instructions, and complete task training and practice.

The Levenson Psychopathy Scale (Levenson et al., 1995; Lynam et al., 1999; Brinkley et al., 2004), adapted from the Psychopathy Checklist-Revised (PCL-R: Hare, 2003) which was based in part on clinical interviews and shown to be reasonably related to personality assessments (Miller et al., 2008), was used to assess varying levels of psychopathy characteristics in the current sample. The Levenson Scale consists of 26 items that yield two subscales roughly paralleling the two-factor structure of the PCL-R. The primary subscale assesses a “selfish, uncaring and manipulative posture towards others,” while the secondary subscale addresses “impulsivity and a self-defeating lifestyle” (Levenson et al., 1995). We focused on the primary subscale that considers interpersonal/affective features and measures a personality trait rather than behaviors. Clinically relevant levels of psychopathy on this scale are generally designated for those scoring in the upper third, however, here we conceptualized psychopathy as varying continuously along a normal dimension of personality.

Participants without a history of drug abuse, returned to the NIDA-IRP facility on the morning of the PET session, whereas ex-abusers were required to stay overnight to ensure abstinence was maintained. Ex-abusers testing positive for illicit substances were excused from the study. All participants were required to be in the PET facility approximately 2 h prior to task onset to familiarize them with the setting, relax, and interact with clinical staff.

### Task

**Figure 1:**
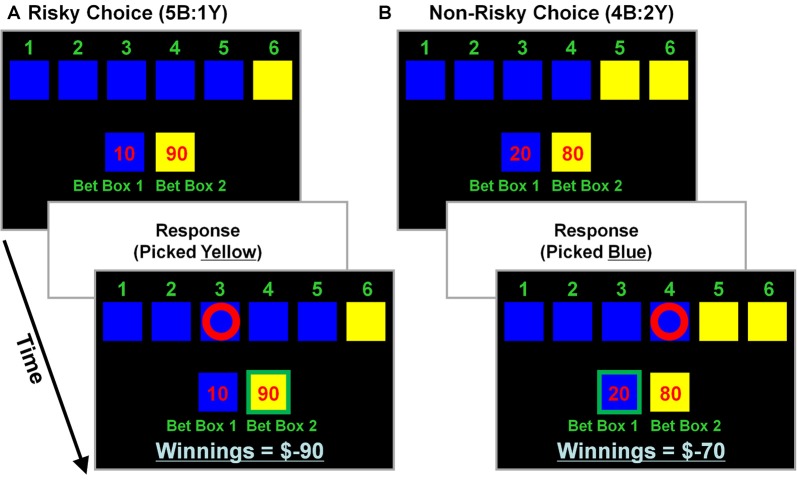
Cambridge decision-making task (CDMT) schematic. Participants were instructed to maximize their accumulated point totals by choosing between one of two options (i.e., pick blue or yellow). Six colored boxes were displayed, in one of which was “hidden” a winning token. The ratio of colored boxes provided information on the odds of the blue (B) and yellow (Y) boxes containing the token (5B:1Y [16.6% chance in yellow], 4B:2Y [33.3% chance in yellow] and 3B:3Y [50% chance in yellow]). The “bet boxes” provided information on the number of points associated with each of the color options (blue:yellow; 10:90, 20:80, 30:70; 40:60, 50:50). Participants selected the color they anticipated contained the hidden token via a button press. After making a selection, feedback was presented, such that a correct selection resulted in the addition of the number of points at stake to the “winnings” total, whereas an incorrect selection resulted in the subtraction of the same amount. Larger rewards were associated with the yellow options which also were less likely to contain the hidden token (except during neutral risk trials [i.e., 3B:3Y]). A risky selection was operationalized as having chosen yellow in the 5B:1Y or 4B:2Y conditions, regardless of the points at stake. **(A)** Example of a risky selection. This trial involves 5 blue boxes and 1 yellow box (5B:1Y) with 10 points at stake associated with a blue selection and 90 points with yellow (10:90). In this example, picking yellow (a risky selection) was an incorrect choice that resulted in subtracting 90 points from the “winnings” total (initially 0 at task onset). **(B)** Example of a non-risky selection. This trial involves 4 blue and 2 yellow boxes (4B:2Y) with 20 points at stake associated with a blue selection and 80 points with yellow. In this example, picking blue (a non-risky selection) was a correct choice that resulted in adding 20 points to the winning total (was −90 from the previous trial).

The CDMT (see also, Rogers et al., 1999a, b; Fishbein et al., 2005) was performed during two separate-task runs to assess the putative link between levels of trait psychopathy and the propensity to make risky decisions. During the task various scenarios were presented that involved “gambling” a certain number of points based on the probability that a given choice would be correct (Figure 1). Participants were instructed that a winning token was hidden in one of six colored (blue or yellow) boxes and to select the color (not the individual box) they believed contained the token via a button press. The ratio of blue (B) to yellow (Y) boxes on a given trial provided information on the odds of winning associated with the two options (5B:1Y; 4B:2Y; and 3B:3Y). Specifically, on the 5B:1Y trials there were 5 blue boxes and 1 yellow box indicating a 16.6% chance that the ring was in the yellow box, the 4B:2Y ratio reflected a 33.3% chance yellow was correct, and the 3B:3Y, a 50% chance. There were also a number of points that appeared in a pseudo-random order for each blue and yellow option (blue:yellow; 10:90, 20:80, 30:70, 40:60, 50:50) that varied independently of the odds ratios. A correct choice resulted in the addition of the number of points associated with that outcome, while an incorrect choice resulted in the subtraction of the same amount. Larger rewards were always associated with the yellow options and, because the yellow options were the less likely correct-outcome (except during the neutral risk scenarios, i.e., 3B:3Y), these were considered risky selections. Thus, the inherent conflict in the task was that the less likely options were generally associated with larger numbers of points to be gained or, more often, lost. A risky-choice was operationalized as selecting the yellow option in the 5B:1Y and 4B:2Y conditions. On each trial of the task participants were given an indefinite amount of time to make a selection. Behavioral performance metrics included the percent of risky selections made, total number of points accumulated, and mean reaction times (RTs). No monetary value was attached to accumulated points. Each run of the CDMT lasted ~8 min.

Participants performed two separate CDMT runs each consisting of 75 trials (25 trials/ratio). PET images were acquired (see below) while participants completed an embedded sequence of 21 trials considered to be of the highest risk by virtue of containing the least likely options with high point values (e.g., 5B:1Y associated with 10:90 points; 5B:1Y with 20:80 points; 5B:1Y with 30:70 points; 4B:2Y with 10:90 points; 4B:2Y with 20:80 points; 4B:2Y with 30:70 points). The 21-trial scanning sequence lasted at least 100 s (the total duration was a function of the participants’ RTs), encompassing the PET image acquisition period. This embedded sequence aided in the ability to acquire PET images during performance of roughly comparable trials across participants.

Two separate runs of a sensorimotor control task were also performed that required attention and motor responses, but did not involve decision-making based on task contingencies. In the control task, one of two displayed boxes changed color and participants indicated with a button press the color of the box that had changed. A total of 80 trials were presented during the control task runs which lasted ~3.5 min each.

### Behavioral Analyses

Behavioral data were analyzed in SPSS 20 (Chicago, IL, USA). Behavioral measures (percent risky decisions, points accumulated and RT) were initially assessed in a repeated-measures ANCOVA framework to assess for differential relations between CDMT performance metrics in Run 1 vs. Run 2 and the continuous psychopathy scores. Specifically, we were interested in the RUN × PSYCHOPATHY interaction effect which indicated if the relationship between the behavioral measure and psychopathy scores differed as a function of run. Given that psychopathy scores were related to DUS (*r*_(27)_ = 0.59, *p* = 0.001) and sex (*t*-test comparing males vs. females: *t*_(26)_ = 2.4, *p* = 0.023; Supplementary Figure S1), these variables were controlled for in the ANCOVA model and all subsequent analyses. We then conducted follow-up partial correlation analyses separately for Run 1 and Run 2 behavioral measures to further characterize the relation between CDMT performance and psychopathy. When conducting these partial correlation analyses for Run 2, we further controlled for the Run 1 behavioral measure, which allowed for assessment of change related to task performance (i.e., analogous to a Run 2 minus Run 1 difference score).

### Image Acquisition

PET image acquisition and reconstruction procedures were the same as those employed by Ernst et al. (2002), Bolla et al. (2003) and Fishbein et al. (2005). Images were acquired on a Siemens ECAT EXACT HR+ scanner in 3D mode. Prior to scan acquisition, participants were positioned on the scanner bed and fitted with a plastic mask (Tru-Scan Imaging, Inc., Annapolis, MD, USA) to minimize head movement during data collection. A 20-min transmission scan was acquired using rotating ^68^GE rod sources before radiotracer administration. Image acquisition involved five separate 370 MBq injections of H_2_^15^O and consisted of one baseline and four task-related measurements; two each for the CDMT and control task. During the CDMT, the embedded 21-trial scanning sequence began ~60–90 s after task onset. PET acquisition scans were 60 s each. The order of CDMT and control task runs was counterbalanced across participants. Images were reconstructed by applying corrections for attenuation and scatter using a Hann filter (cut-off frequency: 0.5 cycles/pixel) and filtered back projection algorithm. Image resolution following reconstruction was 6.52 and 7.16 mm in the x and y transverse planes, respectively, at the field of view center, and 3.72 in the axial plane.

### Image Processing and Analyses

Processing and analyses of PET images were performed using Statistical Parametric Mapping software (SPM99 and SPM5, Wellcome Department of Cognitive Neurology, London, UK). Preprocessing in SPM99 included: realignment to correct for head motion, spatial normalization using the Montreal Neurologic Institute (MNI) template, and spatial smoothing with a standard 12 mm full-width at half-max Gaussian kernel. Subsequent imaging analyses were performed with SPM5.

#### Task and Run Effects

To identify brain regions associated with CDMT performance, the overall TASK effect was examined by first creating contrast images (2 CDMT minus 2 control task images) for each participant using grand mean scaling with ANCOVA normalization. These contrast images were subsequently analyzed in a second-level voxel-wise one-sample *t*-test including psychopathy score, DUS, sex and task-related behavioral measures (percent risky selections made, points accumulated, and RT) as covariates. Significant task-related activation clusters (CDMT > control task) were identified using a *p*_cluster-corrected_ < 0.05 threshold (*p*_voxel-level_ < 0.001, cluster-extent 167 voxels, determined via AFNI’s 3dClustSim program). To identify differences in rCBF associated with individual RUNS, contrast images were separately created for Run 1 (CDMT-R1 minus control-R1) and Run 2 (CDMT-R2 minus control-R2) for each participant. These contrast images were analyzed in separate second-level one-sample *t*-tests employing the aforementioned run-specific covariates and thresholds.

#### Psychopathy-Related Effects

To identify brain regions showing correlations between rCBF and trait psychopathy during CDMT performance, we assessed the statistical parametric maps specifically associated with the continuous psychopathy variable in the one-sample *t*-test model described above (which also contained the DUS, sex and task-related behavioral measure variables). Significant clusters showing correlations (positive or negative) with psychopathy scores were identified using a *p*_cluster-corrected_ < 0.05 threshold (*p*_voxel-level_ < 0.001, cluster-extent: 167 voxels).

## Results

### Behavioral Measures

**Figure 2:**
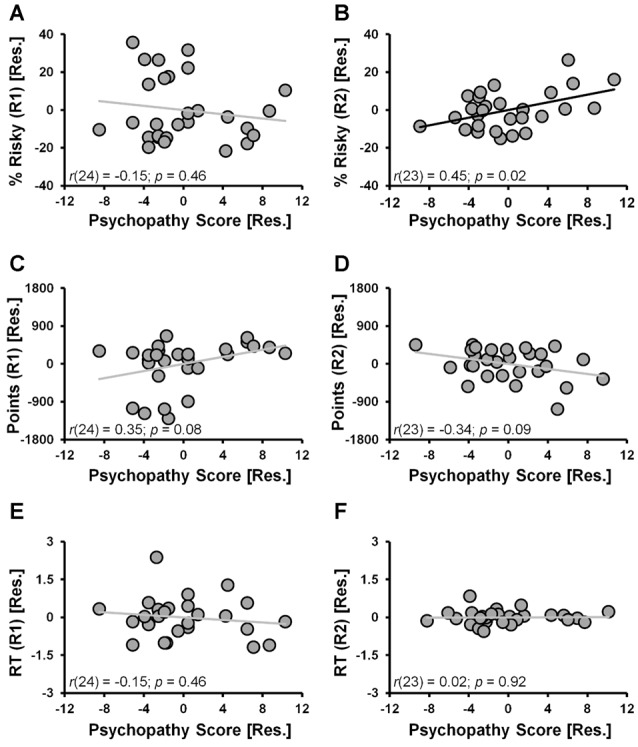
CDMT behavioral outcomes as a function of psychopathy scores. **(A,B)** Trait psychopathy scores were differentially correlated with the percent of risky selections (% Risky) made in Run 1 **(A)** vs. Run 2 **(B)** (RUN × PSYCHOPATHY: *p* = 0.024). Whereas during Run 1 the correlation failed to reach significance (*p* = 0.46), during Run2, risky selections were positively correlated with psychopathy scores (*p* = 0.02). **(C,D)** Trait psychopathy scores were differentially correlated with the total number of points accumulated in the two task runs (RUN × PSYCHOPATHY: *p* = 0.016). Although failing to reach significance, during Run 1 there was a positive relation between points accumulated and psychopathy (*p* = 0.08), whereas during Run 2 this correlation was negative (*p* = 0.09). **(E,F)** Trait psychopathy scores were not related to reaction times (RTs) from either Run 1 **(E)** or Run 2 **(F)** of the task. To facilitate visual interpretation of the partial correlations, the scatter plots depict residualized values for the variables shown on the x- and y-axes (indicated by [Res.]) where the influences of the controlled variables have been removed (scatter plots of the raw values can be found in Supplementary Figure S2). Black best fitting lines indicate a statistically significant partial correlation and gray lines indicate associations that failed to reach significance.

When considering the percent of risky decisions made, a significant main effect of RUN (*F*_(1,24)_ = 13.2, *p* = 0.001) in the ANCOVA model indicated that risky selections generally decreased from Run 1 (24.1% ± 3.4%) to Run 2 (18.1% ± 2.6%) of the task. A significant RUN × PSYCHOPATHY interaction effect (*F*_(1,24)_ = 5.8, *p* = 0.024, controlling for DUS and Sex) indicated that psychopathy scores were differentially correlated with the percent of risky selections made in Run 1 vs. Run 2. Specifically, during Run 1 the partial correlation between risky selections and psychopathy scores failed to reach significance (Figure 2A; *r*_(24)_ = −0.15, *p* = 0.46; controlling for DUS and sex); whereas during Run 2, risky selections were positively correlated with psychopathy scores (Figure 2B; *r*_(23)_ = 0.45, *p* = 0.02; controlling for DUS, sex and Run 1 risky selections)^1^. These outcomes indicate that higher levels of psychopathy, above and beyond that attributable to DUS and sex, were associated with greater tendencies to make risky selections only in the second half (i.e., Run 2) of the task.

The selection of risky options was a disadvantageous strategy in the CDMT leading to smaller accumulated point totals as indicated by an inverse relation between the total percent of risky selections made and total points accumulated (*r*_(27)_ = −0.69, *p* < 0.001). Paralleling the percent risky selection outcomes, a significant main effect of RUN (*F*_(1,24)_ = 6.8, *p* = 0.015) indicated that the number of points accumulated generally increased from Run 1 (500.4 ± 105.9) to Run 2 (512.5 ± 72.6). A significant RUN × PSYCHOPATHY interaction effect (*F*_(1,24)_ = 6.7, *p* = 0.016), indicated that psychopathy scores were differentially correlated with the total number of points accumulated in Run 1 vs. Run 2 of the CDMT. Although the follow-up partial correlations failed to reach significance, during Run 1 there was a positive relation between points accumulated and psychopathy (Figure 2C; *r*_(24)_ = 0.35, *p* = 0.08; controlling for DUS and sex) and a negative relation between these two variables during Run 2 (Figure 2D; *r*_(23)_ = −0.34, *p* = 0.09; controlling for DUS, sex and R1 points). These outcomes suggest that a greater tendency to make risky decisions by individuals self-reporting higher degrees of psychopathy was a suboptimal task strategy.

With respect to RT, neither the RUN (*p* = 0.2) nor RUN × PSYCHOPATHY effects (*p* = 0.6) were significant. Furthermore, the partial correlations were not significant when considering the relations between psychopathy scores and Run 1 RT (Figure 2E; *r*_(24)_ = −0.15, *p* = 0.46, controlling for DUS and sex) or Run 2 RT (Figure 2F; *r*_(24)_ = 0.02, *p* = 0.92, controlling for DUS, sex and Run 1 RT). These null outcomes suggest that the decision-making strategy employed by individuals with higher self-reported levels of psychopathy was not driven by a failure of inhibitory control involving impulsive, prematurely initiated responses.

### Imaging Results

#### Task Effect

**Figure 3:**
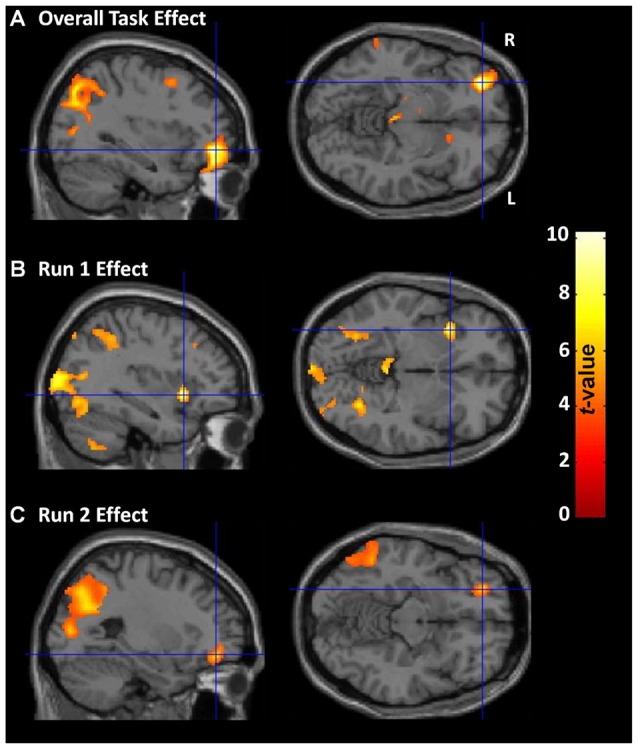
CDMT PET outcomes. **(A)** Overall CDMT (i.e., both Run 1 and Run 2) performance was associated with increased regional cerebral blood flow (rCBF) in expected brain regions notably including the right lateral OFC and bilateral dorsal parietal regions. **(B)** Run 1 CDMT performance was associated with increased rCBF notably in the right insula and bilateral ventral parietal regions. **(C)** Run 2 CDMT performance was associated with increased rCBF notably in the lateral OFC and bilateral dorsal parietal regions. The images are the thresholded (*p*_cluster-corrected_ < 0.05; *p*_voxel-level_ < 0.001) statistical parametric maps resulting from a one-sample *t*-test performed on the subject-level contrast images between the active (CDMT) minus the control (sensorimotor) task when controlling for psychopathy score, drug use severity (DUS), sex, and task performance metrics. See Supplementary Tables S1, S2 for coordinates of task-related brain clusters.

Across all participants and both runs, overall CDMT performance was associated with increased rCBF in regions (Figure 3A, Supplementary Table S1) similar to those identified in previous PET reports (e.g., Rogers et al., 1999b; Ersche et al., 2005). Expected task-induced increases were observed notably in the right lateral OFC, middle frontal gyrus, ACC, bilateral dorsal parietal areas (inferior/superior parietal lobule) and cerebellum. Additional regions showing increased rCBF not observed in Rogers et al. (1999b) included the right dlPFC, bilateral thalamus, and left caudate. Regions previously reported to show increased rCBF during CDMT performance (Rogers et al., 1999b) but absent in the current TASK effect included the right insula/inferior frontal and ventral parietal regions (but, see RUN effects below). Thus, the TASK effect analysis largely replicated results from previous PET studies.

#### Run Effects

Activation patterns were examined separately for each task run to identify qualitative differences in brain function possibly reflecting evolving task strategies. During Run 1, increased rCBF was observed in the right insula/inferior frontal gyrus, bilateral ventral parietal regions (fusiform and middle occipital gyrus), and in similar regions identified from the TASK effect above including: bilateral cerebellum, right inferior parietal lobule, left superior parietal lobule, and right dlPFC (Figure 3B, Supplementary Table S2). In contrast, during run 2 (Figure 3C, Supplementary Table S2), rCBF increases were observed primarily in the right lateral OFC/middle frontal gyrus and dorsal parietal regions. A direct quantitative comparison of Run 1 vs. Run 2 contrast images failed to identify any regions showing significantly different rCBF between runs. These RUN effects suggest subtle differences in brain regions associated with performance as experience with the CDMT progressed.

#### Psychopathy-Related Effects

**Figure 4:**
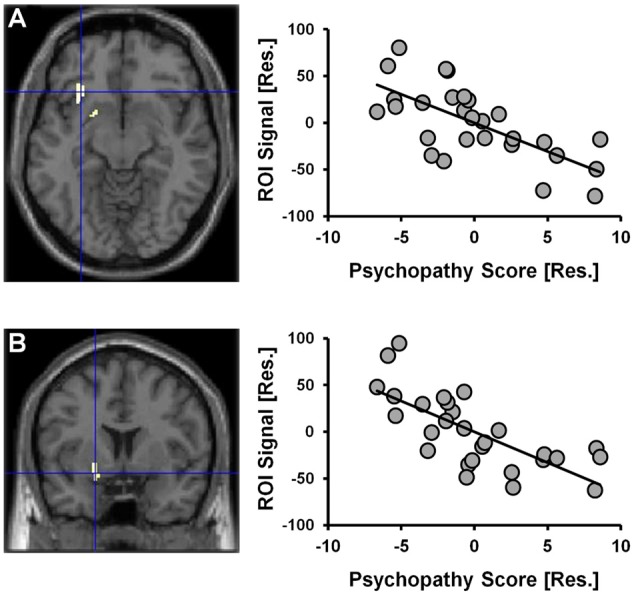
CDMT PET outcomes as a function of psychopathy scores. Psychopathy scores were negatively correlated with rCBF in the right insula **(A)** and the right ventral striatum **(B)** during the CDMT. The images are the thresholded (*p*_cluster-corrected_ < 0.05; *p*_voxel-level_ < 0.001) statistical parametric maps and depict the negative relation between the psychopathy score variable included in the one-sample *t*-test model from Figure 3C showing Run2-related effects (this model also controlled for DUS, sex and task performance metrics). To facilitate visual interpretation, the scatter plots depict residualized values for the variables shown on the x- and y-axes (indicated by [Res.]) where the influences of the controlled variables have been removed (scatter plots of the raw values can be found in Supplementary Figure S3). See Table 2 for coordinates of trait psychopathy-related brain clusters.

Given that a significant positive correlation between psychopathy scores and risky selections became apparent in the latter-half of the CDMT, assessment of psychopathy-related rCBF modulations initially focused on Run 2. When considering the statistical parametric map associated with the psychopathy score variable included in the one-sample *t*-test model used to identify Run 2 related brain activations, psychopathy scores negatively correlated with rCBF in the right insula and the right ventral striatum (Figure 4, Table 2). These areas have been implicated in the integration of cognitive and visceral states (e.g., Bechara et al., 2000; Craig, 2009) and reward processing (e.g., Haber, 2011). We note that these outcomes were observed in a model also controlling for DUS, sex and behavior measures. No significant clusters were observed when considering the positive correlation between psychopathy and rCBF in Run 2 or when considering positive or negative correlations for Run 1 or overall task-related rCBF. Thus, an inverse relation between psychopathy scores and rCBF was only detected during Run 2, that is, when the influence of psychopathy on the percent of risky selections was most apparent.

**Table 2:** Brain regions negatively correlated with trait psychopathy scores during cambridge decision-making task (CDMT) performance.

Brain region	Side	Activation peak (x, y, z, in mm)	Brodmann area(s)	Volume (# voxels)
**Psychopathy score negative correlation**				
A Insula	R	32, 18, 10	13	223
B Ventral striatum	R	20, 6, −12	n/a	167

*Note. See also Figure 4*.

## Discussion

This secondary data analysis study explored the neural correlates of the psychopathy-related propensity to maintain maladaptive decision-making among participants with varying levels of self-reported psychopathy. Participants performed two runs of the CDMT during H_2_^15^O PET scanning to test the general premise that maladaptive decision-making is related to a motivational imbalance characterized by reduced cognitive control and/or feedback evaluation and, concomitantly, heightened reward sensitivity. Behaviorally, we observed a positive correlation between psychopathy scores and the percent of risky selections made during the second run of the task. Neurobiologically, during CDMT performance we observed increased rCBF in the lateral OFC, middle frontal gyrus, insula, ACC, bilateral parietal areas, and the cerebellum, largely replicating the results of previous PET (Rogers et al., 1999b; Ersche et al., 2005) and fMRI studies (Ernst and Paulus, 2005; Krain et al., 2006). Regarding psychopathy-related relations with brain measures during CDMT performance, the outcomes partially supported our hypotheses. Specifically, as hypothesized, we observed that right insula rCBF was negatively correlated with psychopathy scores, yet contrary to our hypothesis, striatal rCBF was negatively correlated (as opposed to a hypothesized positive relation) with psychopathy scores.

As anticipated, participants reporting higher levels of psychopathy generally made more risky selections in the second run of the task. This finding suggests that those with higher trait psychopathy were less likely to adopt a more advantageous decision-making strategy as experience with the task in general and negative feedback in particular increased. The continued selection of risky options is generally consistent with previous studies (Mitchell et al., 2002; van Honk et al., 2002; Dean et al., 2013), further suggesting that psychopathic tendencies may be associated with an inability to translate experience with negative outcomes into behavioral adaptations. Many have surmised that the psychopathy-related inability to adopt optimal decision-making strategies is indicative of attenuated somatic/emotional signals which normally function to guide avoidance of negative outcomes (Bechara et al., 2000; van Honk et al., 2002; Bechara, 2004; Birbaumer et al., 2005; Hughes et al., 2016; Snowden et al., 2017). Neuroimaging studies aid in this interpretation as the neuroanatomy of somatic signaling and reward processing has been well delineated.

PET outcomes paralleled the behavioral data in that correlations between rCBF and psychopathy scores were only detected during the second task run. Specifically, higher psychopathy scores were correlated with reduced functional activity in right-lateralized insular and ventral striatal regions. Decreased activation in those with higher self-reported psychopathy is suggestive of an impaired ability to detect the need for, and/or the actual deployment of, increased cognitive control and a tendency to seek reward undeterred by somatic states signaling the higher probability of imminent penalties. Consistent with this interpretation, the insula has been implicated in an affective monitoring role that relates actions to their interoceptive consequences (Brass and Haggard, 2010). The awareness of somatic changes (Critchley et al., 2004; Craig, 2009) instantiated by the insula or the reactivation of somatic marker representations (Bechara, 2004) in regions connected with the insula, including the ventral striatum, has been proposed to guide future decisions by endowing actions with subjectively-experienced reinforcement or punishment value (Bechara et al., 2000). The observed correlation within the insula suggests that participants with higher levels of trait psychopathy were less efficient at mobilizing affective resources necessary for full integration of action outcomes with interoceptive consequences.

Based on previous studies, such as those relating elevated mesolimbic dopamine responsivity to rewards with higher-trait psychopathy (Buckholtz et al., 2010), we originally hypothesized that psychopathy scores would positively correlated with rCBF in the striatum. While we were able to provide evidence supporting the notion that the psychopathy-related propensity to maintain risky decision-making in spite of diminishing returns may be a function of impaired feedback evaluation and/or attenuated cognitive control, evidence for increased reward sensitivity requires further scrutiny. The striatum has been the focus of many psychopathy-related studies given its role in integrating information from cognitive, affective, and sensory systems, as well as the potential for delineating the somatic marker premise in the context of risky decision-making (see Yildirim and Derksen, 2013 for review). Feedback loops from affective systems to the striatum function to maintain and prioritize attention toward attainable goals, such as the relative prospects of receiving either rewards or punishment. Thus, in anticipation of reward, its activation increases approach behavior, and if penalties are forthcoming, its activation leads to withdrawal or flight from the stimulus (Glenn and Yang, 2012). Inhibiting response tendencies under either of these circumstances is associated with deactivation of the striatum. Many investigators, particularly those considering the somatic marker hypothesis in psychopathy, have anticipated that the striatum would be hypersensitive to rewards, giving rise to a heightened sense of salience for rewarding stimuli and a relative disregard for, in many cases, a higher probability of negative consequences. Studies have not, however, unilaterally found evidence for these expectations. For example, Glenn and Yang (2012) postulated that the striatum among psychopaths may be ineffectively processing the lack of a reward after repeated negative feedback, leading to continuous responding to a stimulus that is not resulting in a reward and may be instead more often associated with penalties. Also of note, reduced striatum activity may be indicative of cortico-striatal dysfunction (Hosking et al., 2017); further studies using resting-state fMRI would help delineate differences in individuals with higher-trait psychopathy within precise brain networks.

Several limitations of the current exploratory study of psychopathic traits are noteworthy. First, psychopathy scores were related to past drug use which was statistically controlled for in the behavioral and neuroimaging analyses. Nonetheless, by simply including a measure of DUS as a covariate, we cannot completely discount the possibility that some of the behavioral and brain correlations with psychopathy scores were tied to past drug use. Chronic drug use has been associated with impaired CDMT performance (Rogers et al., 1999a) and altered brain activity during decision-making (Bolla et al., 2003; Ersche et al., 2005; Fishbein et al., 2005; Gorini et al., 2014). However, other studies have suggested that not all addicted individuals show decision-making impairments (Bechara and Damasio, 2002) and that additional factors, for example, poor working memory (Bechara and Martin, 2004) or psychopathy-related characteristics (Vassileva et al., 2007), may mediate such deficits. Furthermore, rCBF alterations previously reported in drug abusing populations are qualitatively different from the psychopathy-related results herein. Specifically, both Bolla et al. (2003) and Ersche et al. (2005) observed no behavioral differences and increased lateral OFC activity during decision-making in drug-addicted vs. control groups. In cocaine-dependent subjects, Adinoff et al. (2003) reported lower rCBF in the left dlPFC during performance of a gambling task, but not OFC. Gorini et al. (2014), employing a different technique (transcranial direct-current stimulation) identified hypoactivation in the right dlPFC during two risky decision-making tasks in recently abstinent cocaine abusers; stimulation to this region increased selection of non-risky options. Other decision-making studies using fMRI further implicate the OFC in substance abuse (De Bellis et al., 2013; Gowin et al., 2014) and different patterns of activation (e.g., decreased ACC and increased insula activity) among substance addicts (Gowin et al., 2014) than those patterns found in the current investigation focusing on psychopathy traits. For example, when considering only drug-related effects in this current sample, Fishbein et al. (2005) identified an ACC region as significantly different between abstinent drug-user and non-user groups. In short, although DUS correlated with psychopathy scores, previous studies focusing on substance use did not identify similar neural alterations to those we identified as being associated with psychopathy scores. This is consistent with the interpretation that the psychopathy correlations observed herein account for variability in risky decision-making behavior and brain activity above and beyond that attributable to drug use history. Therefore, we suggest that the current results are more in-line with the psychopathy-related literature than that pertaining to drug abuse. Of course, these outcomes are exploratory and future confirmatory studies are needed to further characterize psychopathy-related neural alterations mediating the increased propensity for risky decision-making.

A second limitation pertains to the secondary analytic nature of the current study. The primary study was not optimally designed to examine the impact of psychopathy on brain and behavioral measures and the ability of the current outcomes to generalize to a more appropriately selected sample is unknown. For example, these results are not generalizable to the larger population of individuals with non-forensic psychopathy as the subgroup with active comorbidity (Axis I diagnoses) were excluded to avoid the effects of psychotropic medications commonly used on neural activation. Third, the sample size of this exploratory investigation is small. Also, given the notable paucity of studies including females with psychopathic traits, both male and female participants were included in the current assessment with adjustments for sex. However, the current results should be interpreted with caution as psychopathy scores differed between females and males. Finally, another potential limitation is the lack of monetary compensation offered for points accrued during the CDMT, which may be a disincentive for subjects to perform as well as possible. However, previous studies using similar tasks did not provide monetary rewards and findings were robust (Rogers et al., 1999b; Ersche et al., 2005).

There are a few potential implications of our findings in combination with those of other studies that similarly report differences in striatal and insular cortices among individuals high in psychopathic traits. For example, it may be possible to design interventions to strengthen regulatory processes, known to be malleable in response to environmental inputs (Stuss, 2011; Tracy and Osipowicz, 2011; Venkatakrishnan and Sandrini, 2012). Pharmacological or psychosocial therapies designed to stimulate activity of these structures and their connections (e.g., akin to deep brain stimulation in depression: Drevets et al., 2008) and reinforce prefrontal inhibitory control may normalize emotional regulatory deficits. Another intriguing possibility is the potential preventative effect of educating caregivers, educators and public health policy-makers regarding approaches that may address the developmental pathways of youth expressing psychopathic, or relatedly, callous-unemotional traits. For example, early enrichment, tactile stimulation, stress reduction and other environmental enhancements early in life may strengthen prefrontal cognitive control and enlarge the striatum to reduce the novelty-seeking and emotional dysregulation associated with psychopathy (Glenn and Yang, 2012). Current therapeutic inefficiencies arise because treatment methods do not map program components to underlying etiologies (Moffitt et al., 2008; Frick and Moffitt, 2010). Targeting program components to subgroups that also confer vulnerability to substance abuse in these individuals are likely to influence responsivity to a given intervention and may substantially improve outcomes. The goal of this line of research is to gain a basic understanding of mechanisms for eventual translation to guide more effective intervention strategies (e.g., assessments to inform clinical case conceptualization for at-risk youth and young adults).

Based on the heuristic framework that the psychopathy-related propensity to maintain disadvantageous decision-making is mediated by altered outcome evaluation, cognitive control, and/or reward sensitivity, we examined the relations between self-reported psychopathy scores and behavioral and brain measures during CDMT performance. Higher levels of psychopathy were associated with a reduced capacity to adapt behavior in concordance with feedback. In parallel, we observed that higher psychopathy scores correlated with reduced activity in the insula and striatum which may underlie decreased efficiency integrating cognitive and affective processes to inform decision-making strategies for performance optimization. Although PET provides a blunt neuroimaging tool to dissect the complex decision-making process, based on the anatomical similarity between regions showing reduced activity in higher trait psychopathy individuals with those in previous cognitive control investigations, we speculate that poor psychopathy-related decision-making may be at least partly mediated by the attenuated deployment of top-down cognitive control resources particularly following negative action outcomes. Thus, these results may be useful for the design of future confirmatory event-related fMRI studies interrogating the psychopathy-related neural substrates of risky decision-making.

## Author Contributions

DHF was the prime on the grant award, conceived the study, and contributed to the writing. MTS conducted the data analysis and contributed writing to each section of the article.

## Conflict of Interest Statement

The authors declare that the research was conducted in the absence of any commercial or financial relationships that could be construed as a potential conflict of interest.

## Footnotes

^1^From a slightly different perspective, the Run 2 minus Run 1 difference score for percent risky selections (where a positive difference score indicates more risky decisions in Run 2) was positively correlated with psychopathy scores (*r*_(24)_ = 0.44, *p* = 0.02, controlling for DUS and sex).
