# Supplementary material for: Higher Trait Psychopathy Is Associated with Increased Risky Decision-Making and Less Coincident Insula and Striatal Activity
Source: Front Behav Neurosci. 2017 Dec 12;11:245. doi: 10.3389/fnbeh.2017.00245 (PMC5732997; doi:10.3389/fnbeh.2017.00245)
Supplement: Supplementary file 1 [file Presentation_1.pdf]

SUPPLEMENTAL INFORMATION

Higher Trait Psychopathy is associated with increased risky decision-making and less coincident insula and striatal activity

Matthew T. Sutherland and Diana H. Fishbein

SUPPLEMENTAL CONTENT

SUPPLEMENTAL Tables

- Table S1: Coordinates of CDMT-related brain activation (TASK effect)
- Table S2: Coordinates of CDMT-related brain activations (RUN effects)

SUPPLEMENTAL FIGURES

- Figure S1: Relation between drug use severity and sex with psychopathy scores
- Figure S2: CDMT behavioral performance outcomes (raw variable values)
- Figure S3: CDMT PET outcomes (raw variable values)

Table S1. Regions showing increased rCBF during CDMT performance.

	Brain Region	Side	Activation Peak (x, y, z, in mm)	Brodmann area(s)	Volume (# voxels)
1	Middle frontal gyrus/lateral OFC	R	32 48 -8	10/11	1678
2	Cingulate gyrus	R	14 26 30	32/9	1378
3	Middle frontal gyrus/dlPFC	R	50 28 34	9/46	378
4	Middle frontal gyrus	R	20 -4 60	6	273
5	Inferior parietal lobule	R	40 -52 38	7/40	6598
6	Inferior parietal lobule	L	-34 -54 50	7/40	2021
7	Superior parietal lobule	L	-18 -72 56	7	302
8	Inferior temporal gyrus	R	6 -52 -26	20/37	272
9	Cerebellum	L	-36 -70 -26	lateral	798
10	Cerebellum	L	-2 -78 -44	middle	317
11	Cerebellum	L	-46 -58 -44	lateral	376
12	Thalamus	L	-6 -26 14	--	198
13	Thalamus	R	16 -16 -4	--	753
14	Caudate	L	-16 18 -4	--	283

Note. See also main text, Fig. 3A

Table S2. Regions showing increased rCBF during Run1 and Run2 of the CDMT.

	Brain Region	Side	Activation Peak (x, y, z, in mm)	Brodmann area(s)	Volume (# voxels)
**Run 1**					
1	Inferior frontal gyrus/Insula	R	34 16 -4	47	335
2	Middle frontal gyrus/dlPFC	R	38 28 40	9/8	403
3	Middle occipital gyrus/fusiform	R	28 -76 10	30/31/18/19	2780
4	Fusiform	L	-32 -76 -20	18/19	2357
5	Inferior parietal lobule	R	38 -46 42	40	583
6	Superior parietal lobule	L	-24 -76 46	7/19	212
7	Cuneus	L	-12 -80 12	18/17	1541
8	Cerebellum	R	4 -72 -28	middle	921
9	Cerebellum	R	22 -44 -50	tonsil	256
10	Cerebellum	L	-42 -50 -46	lateral	309
11	Caudate	L	-10 6 16	---	235
**Run 2**					
1	Middle frontal gyrus/Lateral OFC	R	26 44 -12	11/47/10	454
2	Superior parietal lobule	R	30 -76 44	7/19	3964
3	Fusiform	R	54 -68 -18	19/37	754
4	Precueneus	L	-22 -80 36	19/7	1215

Note. See also main text, Fig. 3B and 3C.

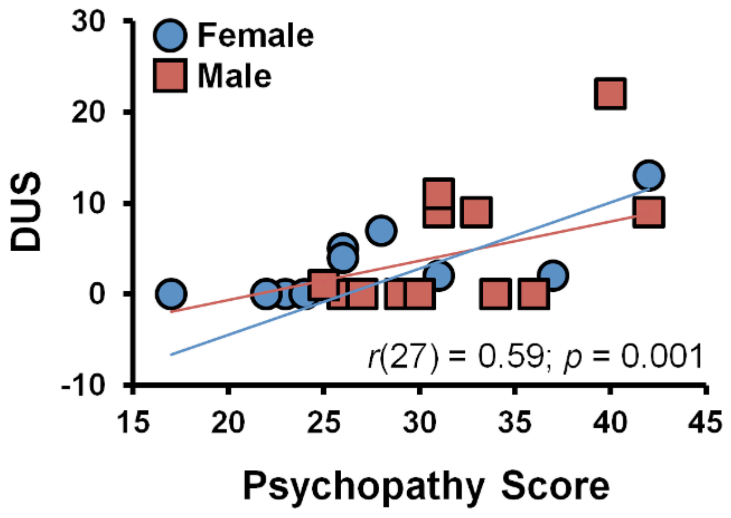

Figure S1. Relations between trait psychopathy scores, drug use severity (DUS), and sex. The current secondary analysis of an existing data set, explored the relation between self-reported psychopath and behavioral and brain metrics during a risky decision-making task. While the previous report of these data (Fishbien et al., 2005) was optimally designed to examine the influence of drug use on such metrics, the study was not optimal for examining the influence of psychopathy (hence the exploratory designation of this report). As depicted in the scatter plot, psychopathy scores were also related to DUS and sex. Specifically, psychopathy scores were positively correlated with DUS ratings across all participants ($r[27] = 0.59$, $p = 0.001$) and were significantly higher among male (red; 32.1 ± 1.3) relative to female participants (blue; mean = 26.9 ± 1.7) ($t[26] = 2.4$, $p = 0.023$). As such, the influence of DUS and sex was statistically controlled for in all behavioral and brain analyses.

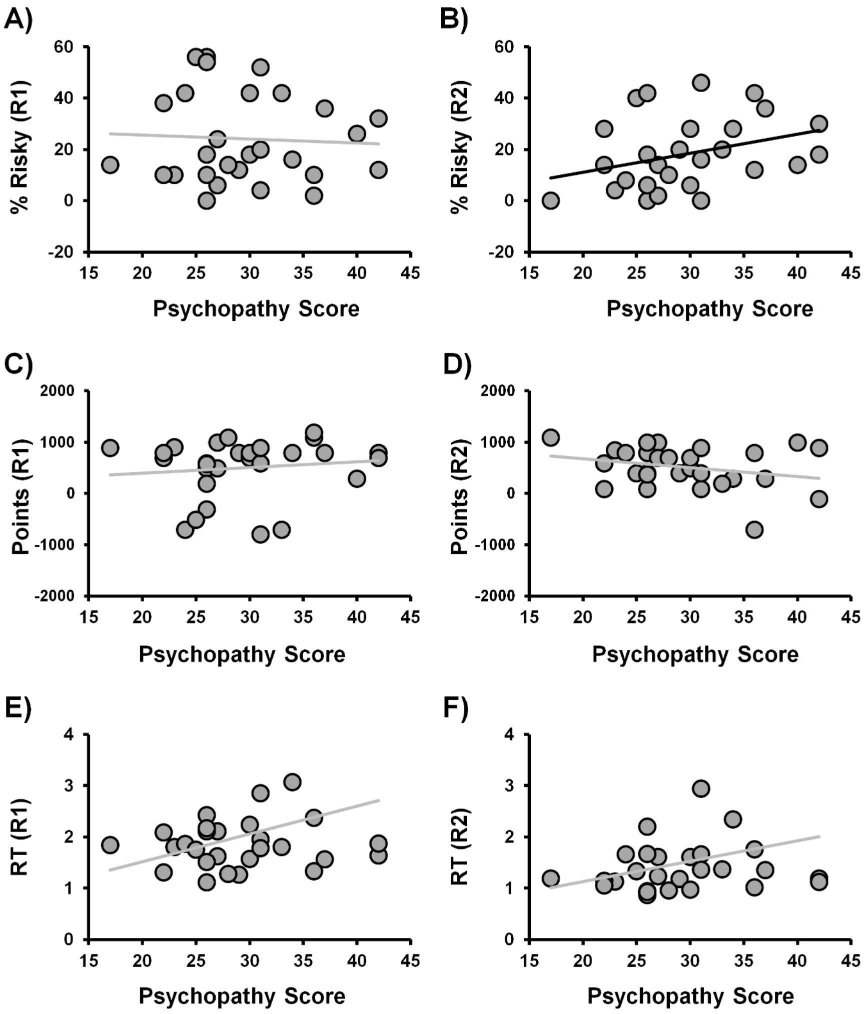

Figure S2. CDMT behavioral performance outcomes (raw variable values). Whereas Figure 2 of the main text displays scatter plots depicting the residulaized values for the variables shown on the x- and y-axes, the scatters here show the variables raw (un-residualized) values. (**A**) Relation between percent risky selections (% Risky) in Run 1 of the CDMT and trait psychopathy scores. (**B**) Relation between percent risky selections (% Risky) in Run 2 of the CDMT and psychopathy scores. (**C**) Relation between total accumulated points in Run 1 of the CDMT and psychopathy scores. (**D**) Relation between total accumulated points in Run 2 of the CDMT and psychopathy scores. (**E**) Relation between reaction times (RT) in Run 1 of the CDMT and psychopathy scores. (**F**) Relation between reaction times (RT) in Run 2 of the CDMT and psychopathy scores.

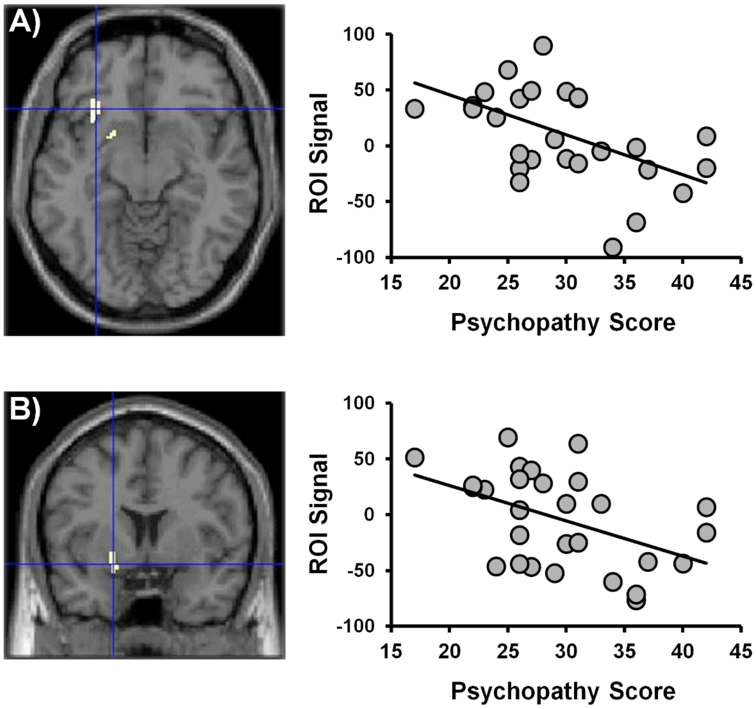

Figure S3. CDMT PET outcomes (raw variable values). Whereas Figure 4 of the main text displays scatter plots depicting the residulaized values for the variables shown on the x- and y-axes, the scatters here show the variables raw (i.e., un-residualized) values. Psychopathy scores were negatively correlated with rCBF in the right insula (**A**) and the right ventral striatum (**B**) during Run 2 of the CDMT.
